# Impact of contrast-enhanced ultrasound optimized breast imaging reporting and data system category on the biopsy decision for non-mass breast lesions

**DOI:** 10.3389/fonc.2026.1731762

**Published:** 2026-02-23

**Authors:** Xuexue Chen, Mengyuan Fang, Huajie Pei, Lingling Li, Bing Zhang, Haimei Lun, Liu Wei, Bing Ling, Yingying He, Qiao Hu

**Affiliations:** 1 People’s Hospital of Guangxi Zhuang Autonomous Region, Department of Ultrasound, Nanning, China; 2 Changsha Hospital for Maternal & Child Health Care Affiliated to Hunan Normal University, Department of Ultrasound, Changsha, China

**Keywords:** biopsy, breast imaging reporting and data system category, contrast-enhanced ultrasound, conventional ultrasound, non-mass breast lesions

## Abstract

**Objective:**

To investigate the impact of contrast-enhanced ultrasound (CEUS) optimized Breast Imaging Reporting and Data System (BI-RADS) prediction model on the biopsy decision for non-mass breast lesions (NMLs).

**Methods:**

148 NMLs histopathologically confirmed from 142 patients who underwent ultrasound (US) and CEUS examination were retrospectively enrolled. US and CEUS features were compared between malignant and benign NMLs. A CEUS-optimized BI-RADS category prediction model was developed using logistic regression. The diagnostic performance and impact on biopsy decision of US and CEUS-optimized BI-RADS category were compared.

**Results:**

Of 148 NMLs, 77 were malignant and 71 benign. CEUS features such as earlier wash-in, later wash-out, hyperenhancement, heterogeneous enhancement, enlarged enhancement extent, and crab claw-like enhancement were significantly associated with malignancy (P<0.05). The enlarged enhancement extent and crab claw-like enhancement were independent risk factors for malignancy (P<0.05). Using BI-RADS 4B as the cutoff, the sensitivity, specificity, positive predictive value, negative predictive value, and accuracy for diagnosing NMLs increased from 53.2%, 63.4%, 61.2%, 55.6%, and 58.1% before optimization to 93.5%, 81.7%, 84.7%, 92.1%, and 87.8% after CEUS optimization, respectively. With BI-RADS 4A or 4B as the biopsy threshold, the biopsy rate, malignancy detection rate, and missed diagnosis rate were 100%, 52.03%, and 0% before optimization, changing to 75%, 66.67%, and 3.90% after optimization for 4A, respectively. The corresponding values for 4B were 45.95%, 60.29%, and 46.75% before optimization, and 57.43%, 84.71%, and 6.49% after optimization, respectively.

**Conclusion:**

The CEUS-optimized BI-RADS category prediction model could provide valuable guidance for biopsy decision-making in NMLs.

## 1 Introduction

The incidence of breast cancer has been increasing year by year, with its global incidence second only to lung cancer. It is the most common malignant tumor in women and the leading cause of cancer-related death (1). Early detection, timely diagnosis, and prompt treatment are crucial for improved survival and decreased mortality of breast cancer.

Non-mass breast lesions (NMLs) are discrete abnormalities that distinctly differ from normal breast tissue, lack the margination of a mass and cannot be assigned a specific shape. These lesions are often subtle and may visualized primarily in one plane only (2). NMLs accounted for 9.21% of all breast lesions (3). The pathological types of NMLs are diverse. The most common breast cancers identified as non-mass findings on US images were ductal carcinoma *in situ* (DCIS) or invasive lobular carcinoma (ILC) (4). Benign NMLs include fibrocystic change, sclerosing adenosis, radial scar, granulomatous mastitis, and intraductal papilloma, etc (5).

Ultrasound (US) is one of the most commonly used imaging methods for breast cancer screening. The American College of Radiology Breast Imaging-Reporting and Data System (ACR BI-RADS) ^®^ Atlas (6) has described US features of mass-like breast lesions in a standardized format clearly and concisely. However, there’re some limitations when it’s applied to NMLs. Boundaries and margins can be used to determine whether a mass is benign or malignant, while the boundaries of NMLs are often blurred. For example, DCIS is distributed along the duct and the extent of the lesion is difficult to define accurately (7). In such cases, the assessment of the margins is almost ineffective.

Mammography is highly sensitive in detecting microcalcifications. However, it involves radiation exposure and shows relatively low sensitivity for diagnosing dense breast tissue (8). Magnetic resonance imaging (MRI) is widely recognized for its high sensitivity in the diagnosis of breast cancer. However, its clinical utility is often constrained by factors such as high cost, prolonged examination time, and limited sensitivity in detecting microcalcifications. CEUS provides real-time dynamic visualization of microcirculatory perfusion, demonstrating hemodynamic assessment capabilities comparable to MRI (9), while offering advantages in accessibility, shorter examination duration and patient convenience. CEUS is effective in differentiating benign from malignant mass-like breast lesions. However, evidence regarding its application in NMLs remains limited. First, standardized quantitative criteria for CEUS assessment of NMLs are lacking. Second, the value of CEUS in optimizing BI-RADS category for NMLs and its impact on reducing unnecessary biopsies has not been sufficiently investigated. Accordingly, this study aims to systematically investigate the diagnostic value of a CEUS-optimized BI-RADS category prediction model for NMLs and its impact on biopsy decision-making. The specific objectives are as follows: (1) to identify CEUS characteristics for distinguishing benign and malignant NMLs; (2) to evaluate the diagnostic performance of the CEUS prediction model in differentiating NMLs; (3) to explore the role of CEUS in optimizing BI-RADS category of NMLs, thereby providing evidence for reducing misdiagnoses and unnecessary biopsies.

## 2 Materials and methods

### 2.1 Patients

**Figure 1:**
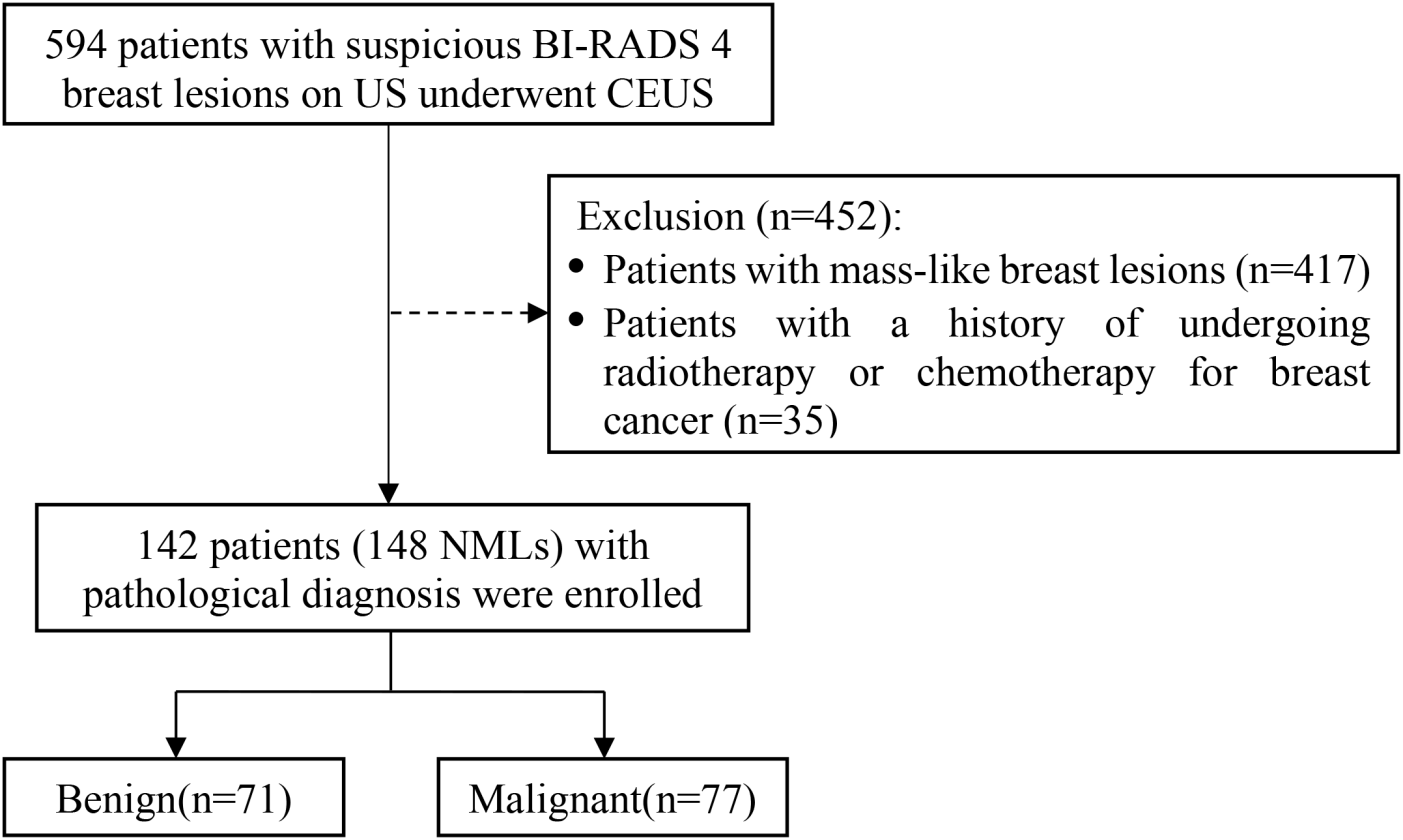
Enrollment flow chart of non-mass breast lesions.

This retrospective study received approval from the Ethics Committee of People’s Hospital of Guangxi Zhuang Autonomous Region (ethical approval number: KY-LW-2020-24). Written informed consent was obtained from all patients. A total of 142 females (aged 29–84 y with a median age of 52 y) underwent US and CEUS examinations were enrolled in the study between March 2021 and April 2025 in the People’s Hospital of Guangxi Zhuang Autonomous Region, where CEUS is employed as a routine diagnostic modality for breast diseases. Selection criteria: (1) The patient underwent CEUS examination with a suspicious BI-RADS category 4 on conventional US; (2) Conventional US features were consistent with the diagnostic criteria for NMLs; (3) All patients underwent both US and CEUS examinations within two weeks before biopsy or surgery; (4) All lesions were pathologically confirmed by either core needle biopsy or surgical excision; (5) Patients had no prior history of breast cancer and had not undergone radiotherapy, chemotherapy, endocrine therapy, or other related treatments. The patient enrollment process was illustrated in **Figure 1**. Among the 142 included patients, 21 underwent both mammography and contrast-enhanced MRI, 21 underwent contrast-enhanced MRI only, and 5 underwent mammography only. The largest lesion diameters varied from 6 to 71 mm, with a mean of (22.7 ± 13.1) mm.

### 2.2 Instruments

Conventional US and CEUS examinations were conducted using the following ultrasound systems: GE Logiq E9 (GE Healthcare, USA), Mindray Resona R9 (Mindray, China), and Vinno Ultimus 9E (Vinno, China). Each system was equipped with linear array transducers operating at a 6–15 MHz frequency range. The ML6-15 (GE), L14-5WU (Mindray), and U5-15L (Vinno) probes were utilized for conventional US. The 9L (GE), L11-3U (Mindray), and U5-15L (Vinno) probes were employed for CEUS.

### 2.3 Conventional ultrasound and CEUS examination

Both conventional US and CEUS examination were performed by the same sonographer with more than 10 years of experience in breast ultrasonography.

Patients were positioned in the supine posture with both arms raised to ensure full exposure of the breasts. Conventional US was used to perform a continuous multi-planar scan of the mammary glands. The following sonographic features were assessed: number, location, size, boundary, shape, aspect ratio, internal echo, posterior acoustic features, microcalcifications, ductal alterations, architectural distortion, and color Doppler flow patterns.

CEUS was performed using the contrast agent SonoVue (Bracco, Italy). A suspension of SF_6_ microbubbles was prepared by reconstituting the lyophilized powder with 5 mL of normal saline, followed by thorough shake. The imaging plane with the most abundant blood supply within the lesion was selected, while including part of the surrounding normal breast tissue for comparison. After switching to CEUS mode with a low mechanical index (0.05-0.10), a 4.8 mL of SonoVue was rapidly injected as a bolus via the antecubital vein, immediately followed by a 5 mL saline flush. A timer was activated simultaneously, and dynamic images were continuously observed and recorded for 2 minutes. During the examination, patients were instructed to remain still and maintain steady breathing to minimize motion artifacts. The selected imaging plane was kept constant, and the transducer was gently placed on the skin to avoid exerting pressure on the lesion.

### 2.4 Image analysis

Two senior sonographers with more than 10 years of experience in breast US, who were blinded to the patient’s information, reviewed the conventional US and CEUS imaging data. They arrived at a consensus by joint review and discussion of disagreements.

According to the US features and the corresponding BI-RADS categories of NMLs suggested by Ko et al. (10), four types could be distinguished. Type I represented duct-like hypoechoic areas without microcalcifications (type Ia) or with microcalcifications (type Ib). Type II represented non-duct-like geographic or mottled areas without microcalcifications (type IIa) or with microcalcifications (type IIb). Type III was associated with architectural distortion. Type IV was associated with posterior acoustic shadowing. NMLs were classified according to their US features as follows: BI-RADS 4A (type IIa), 4B (type Ia, III and IV) and 4C (type Ib and IIb).

Based on previous studies (11, 12), the lesion was compared with the surrounding breast tissue, and the CEUS qualitative features were analyzed, including wash-in/wash-out time (earlier, synchronous, or later), enhancement direction (centripetal, or non-centripetal), enhancement intensity at the peak time (hyperenhancement, isoenhancement, or hypoenhancement), enhancement homogeneity (homogeneous or heterogeneous), enhancement extent (enlarged or not) and crab claw-like enhancement (present or absent).

### 2.5 Statistical analysis

IBM SPSS statistical software version 27.0 was used to perform statistical analysis. Quantitative data are expressed as mean ± standard deviation ( $\bar{x}$ ± *s*). The distribution of variables was analyzed using the K-S test. Groups were compared using the Mann-Whitney U test. The χ^2^-test or Fisher’s exact test was used to evaluate categorical variables. Binary logistic regression analysis was performed to explore the independent risk factors for NMLs. The prediction model was built to optimize the BI-RADS category. Receiver operating characteristic (ROC) curves were constructed to assess the diagnostic performance of the prediction model, and the areas under the ROC curve were compared. The sensitivity, specificity, positive predictive value (PPV), negative predictive value (NPV) and accuracy were also calculated. P<0.05 was considered statistically significant.

### 2.6 Pathology analysis

All patients underwent core needle biopsy (CNB) or surgical excision in two weeks after the ultrasonic examinations. The pathology findings were used as the final diagnostic standard. The choice between core needle biopsy (CNB) and surgical excision was primarily based on a comprehensive assessment of the lesion’s radiologic suspicion for malignancy, size, and clinical factors. Lesions categorized as BI-RADS 4B, 4C, or 5 were generally managed with CNB as the first-line approach. Surgical excision may be considered in the following circumstances: (1) when imaging strongly suggested malignancy, but CNB failed to establish a definitive diagnosis or the pathological findings were discordant with the imaging assessment; (2) for small lesions (≤1 cm) that were easily resectable, or for lesions highly suspected to be benign, such as fibroadenomas, when the patient requested removal; (3) when CNB revealed high-risk lesions, such as atypical hyperplasia or ductal carcinoma *in situ*, surgical excision was necessitated to exclude the presence of invasive carcinoma.

## 3 Results

### 3.1 Patients’ background

The baseline characteristics in the malignant and benign groups was shown in **Table 1**.

**Table 1:** Clinical features of subjects in the malignant and benign groups.

Features	Total (n=148)	Malignant (n=77)	Benign (n=71)	*P* value
Age, mean ± SD, years	52.2 ± 12.3	53.2 ± 12.5	50.5 ± 12.2	0.188
<40, n	23	10	13	0.372
≥40, n	125	67	58	
Pregnancy				
No, n (%)	1	1	0	1.0
Yes, n (%)	147	76	71	
Menopausal status				
Premenopausal, n (%)	85	38	47	0.038
Postmenopausal, n (%)	63	39	24	
Body mass index, mean ± SD, Kg/m²	23.7 ± 3.3	23.6 ± 3.4	23.9 ± 3.2	0.448
<24, n	93	50	43	0.582
≥24, n	55	27	28	
Family history of breast cancer				
No, n (%)	146	75	71	0.513
Yes, n (%)	2	2	0	
Palpable mass in breast				
No, n (%)	50	19	31	0.015
Yes, n (%)	98	58	40	
Nipple discharge				
No, n (%)	141	72	69	0.506
Yes, n (%)	7	5	2	

### 3.2 Pathological data

In total, 148 NMLs were pathologically confirmed by needle biopsy or surgical excision. 77 (52.0%) were malignant, including 28 DCIS, 1 lobular carcinoma *in situ* (LCIS), 29 IDC, 14 IDC with DCIS, 1 IDC with mucinous carcinoma, 3 ILC, and 1 metastatic carcinoma. 71 (48.0%) were benign, including 15 adenosis, 9 sclerosing adenosis, 1 lactational adenosis, 2 lymphocytic mastopathy, 8 fibrocystic mastopathy, 9 fibroadenomas, 1 hamartoma, 1 apocrine adenoma, 1 ductal ectasia, 16 intraductal papilloma, 1 atypical hyperplasia, 6 inflammation, and 1 radial scar.

### 3.3 Ultrasound types of NMLs

Ultrasound Types of NMLs was showed in **Table 2**. The malignancy rate of NMLs with microcalcifications (type Ib and IIb) was 93.55% (29/31), which was significantly higher than those without microcalcifications (P<0.05).

**Table 2:** Ultrasound types of NMLs.

Pathology	No. of lesions	Ultrasound types of NMLs					
		Ia	Ib	IIa	IIb	III	IV
Malignant	77	4	9	36	20	2	6
Benign	71	20	1	44	1	3	2
Total	148	24	10	80	21	5	8
Malignancy rate (%)	52.03%	16.67%	90.00%	45.00%	95.24%	40.00%	75.00%

### 3.4 Analysis of the indicators for malignant NMLs

The CEUS features of NMLs in our study and their correlations with the histopathologic results were outlined in **Table 3**. The results showed that earlier wash-in, later wash-out, hyperenhancement, heterogeneous enhancement, enlarged enhancement extent, and crab claw-like enhancement on CEUS were significantly associated with malignancy (P<0.05).

**Table 3:** Comparison of CEUS qualitative features of benign and malignant NMLs.

CEUS qualitative features		Benign	Malignant	χ^2^ value	P value
Wash-in time	Earlier	25	71	52.654	<0.001
	Synchronous or later	46	6		
Wash-out time	Later	18	64	49.886	<0.001
	Synchronous or earlier	53	13		
Enhancement direction	Non-centripetal	67	65	3.793	0.051
	Centripetal	4	12		
Enhancement intensity	Hypoenhancement or isoenhancement	38	3	45.420	<0.001
	Hyperenhancement	33	74		
Enhancement homogeneity	Homogeneous	42	30	6.030	0.014
	Heterogeneous	29	47		
Enhancement extent	Enlarge	2	47	56.540	<0.001
	Not enlarged	69	30		
Crab claw-like enhancement	Absence	69	35	47.314	<0.001
	Presence	2	42		

**Figure 2:**
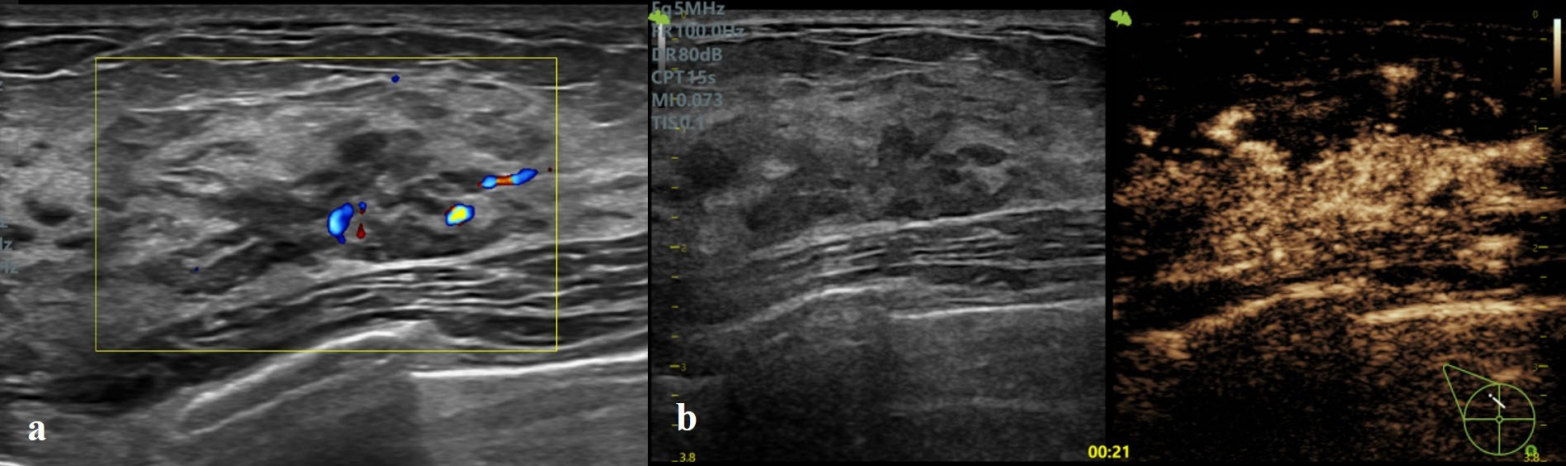
Invasive ductal carcinoma in a 62-year-old female. **(a)** Conventional US showed a duct-like hypoechoic area at 12 o’clock of the right breast, without microcalcifications. **(b)** CEUS showed earlier wash-in, hyperenhancement, contrast retention, enlarged enhancement extent, and crab claw-like enhancement. The lesion was classified as Type Ia and BI-RADS 4B in conventional US and upgraded as BI- RADS 4C after CEUS optimization.

**Figure 3:**
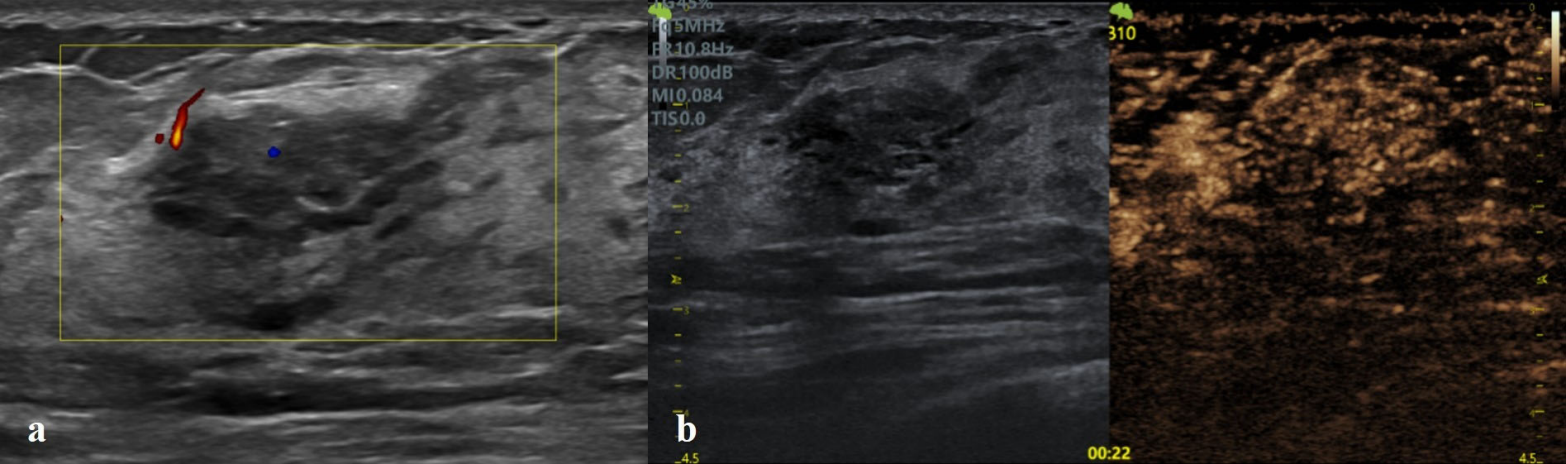
Fibrocystic mastopathy in a 42-year-old female. **(a)** Conventional US showed a non-duct-like hypoechoic area at 10 o’clock of the right breast, without microcalcifications. **(b)** CEUS showed synchronous wash-in, hyperenhancement, earlier wash-out without enlarged enhancement extent and crab claw-like enhancement. The lesion was classified as Type IIa and BI-RADS 4A in conventional US and downgraded as BI- RADS 3 after CEUS optimization.

In the binary logistic regression analysis, the independent risk factors for malignant NMLs were enlarged enhancement extent and crab claw-like enhancement (**Table 4**; **Figures 2**, **3**).

**Table 4:** Logistic regression results of CEUS qualitative features.

CEUS features	β	SE	Wald	P value	OR value	95%CI
Wash-in time	0.661	0.763	0.752	0.386	1.937	0.435-8.639
Wash-out time	0.766	0.666	1.323	0.250	2.151	0.583-7.936
Enhancement intensity	1.019	0.885	1.326	0.250	2.770	0.489-15.702
Enhancement homogeneity	0.780	0.540	2.085	0.149	2.181	0.757-6.282
Enhancement extent	2.767	0.828	11.172	0.001	15.906	3.140-80.563
Crab claw-like enhancement	2.454	0.833	8.687	0.003	11.639	2.276-59.530
Constant	-3.071	0.703	19.105	<0.001	0.046	

### 3.5 Developing the prediction model and evaluation of diagnostic performance

**Figure 4:**
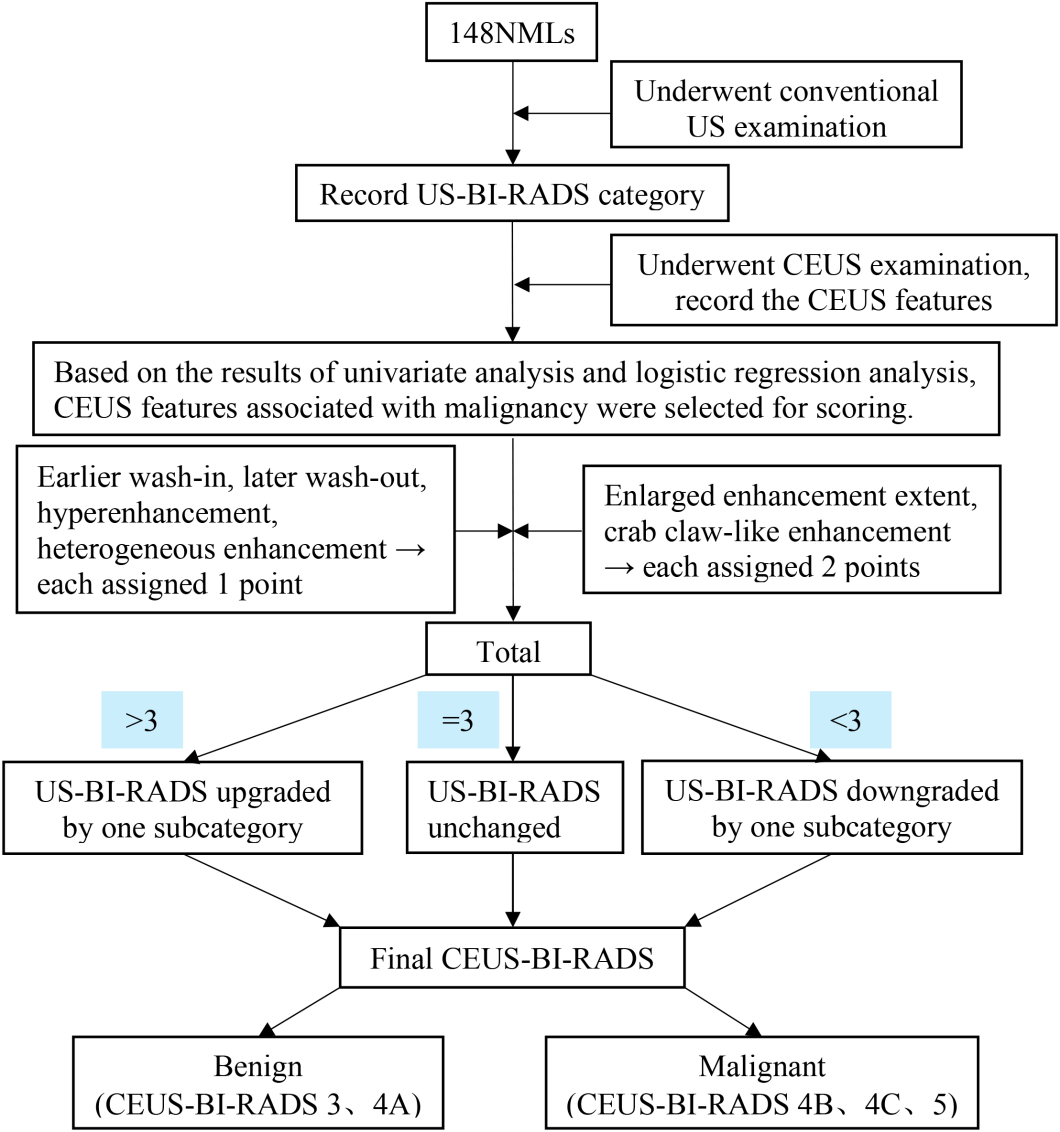
Flowchart of CEUS-optimized predictive model construction.

On the basis of previous studies (13–15) and the indicators for malignant NMLs in the present study, we established a CEUS optimized BI-RADS category prediction model. The CEUS qualitative features associated with malignancy were selected for scoring. Earlier wash-in, later wash-out, hyperenhancement, and heterogeneous enhancement were each assigned 1 point, whereas enlarged enhancement extent, and crab claw-like enhancement were assigned 2 points each. The summation yielded the total score. The BI-RADS category was upgraded by one subcategory when the total score was >3, downgraded when the total score was <3, and unchanged when the total score was 3. The flowchart for constructing the CEUS optimized prediction model was shown in **Figure 4**. The BI-RADS categories before and after CEUS optimization were shown in **Table 5**.

**Table 5:** US and CEUS optimized BI-RADS categories of NMLs.

Methods	Pathology	BI-RADS category				
		3	4A	4B	4C	5
US	Malignant	0	36	12	29	0
	Benign	0	44	25	2	0
	Total	0	80	37	31	0
Malignancy rate (%)		0%	45.00%	32.43%	93.55%	0%
CEUS	Malignant	3	2	34	14	24
	Benign	34	24	11	2	0
	Total	37	26	45	16	24
Malignancy rate (%)		8.11%	7.69%	75.56%	87.50%	100.00%

**Figure 5:**
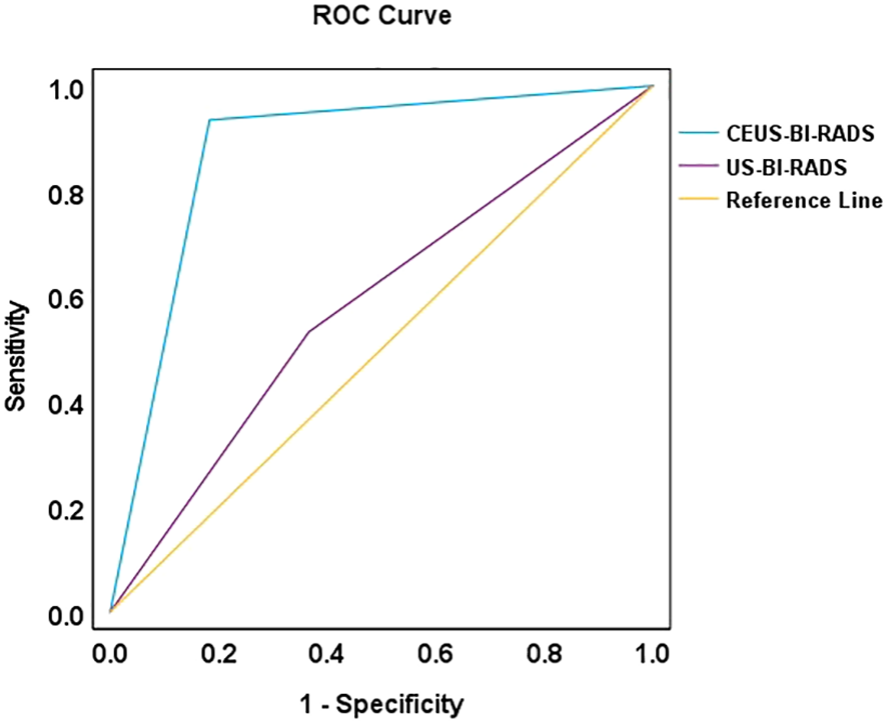
ROC curves of US-BI-RADS and CEUS-optimized BI-RADS.

Using the BI-RADS category as the test variable and pathological malignancy as the state variable, a receiver operating characteristic (ROC) curve was plotted. BI-RADS 4B was used as the diagnostic threshold for malignant NMLs, which corresponded to the maximum Youden index of 0.752. Lesions classified as BI-RADS 3 and 4A were considered benign, whereas those classified as BI-RADS 4B, 4C, and 5 were regarded as malignant. The diagnostic performance of the BI-RADS category in distinguishing benign from malignant NMLs before and after optimization was shown in **Table 6**. Compared to the US-BI-RADS, the CEUS-optimized BI-RADS category demonstrated substantial improvements in the diagnostic performance for NMLs. The sensitivity, specificity, positive predictive value (PPV), negative predictive value (NPV), and accuracy increased from 53.2%, 63.4%, 61.2%, 55.6%, and 58.1% to 93.5%, 81.7%, 84.7%, 92.1%, and 87.8%, respectively(P<0.05). The area under the ROC curve (AUC) improved from 0.583 to 0.876 (P<0.05) (**Figure 5**).

**Table 6:** Diagnostic performance of US-BI-RADS and CEUS-optimized BI-RADS category.

Methods		Pathology		Sensitivity	Specificity	PPV	NPV	Accuracy
		Malignant	Benign					
US	Malignant	41	26	53.2%	63.4%	61.2%	55.6%	58.1%
	Benign	36	45					
CEUS	Malignant	72	13	93.5%	81.7%	84.7%	92.1%	87.8%
	Benign	5	58					

After CEUS optimization,13 benign NMLs were misdiagnosed as malignant, including 1 atypical hyperplasia, 5 intraductal papilloma, 3 inflammation, 2 sclerosing adenosis, and 2 fibrocystic mastopathy. Conversely, 5 malignant NMLs were misdiagnosed as benign, including 4 DCIS and 1 IDC with mucinous carcinoma. BI-RADS category and imaging features of misdiagnosed cases after CEUS optimization was shown in **Table 7**.

**Table 7:** BI-RADS Category and imaging features of Misdiagnosed Cases after CEUS Optimization.

Pathology	No.	US types	US-BI-RADS	CEUS- BI-RADS	CEUS features
atypical hyperplasia	1	IIa	4A	4B	earlier wash-in, later wash-out, heterogeneous hyperenhancement, enlarged enhancement extent, crab claw-like enhancement
intraductal papilloma	4	Ia	4B	4B	earlier wash-in, later wash-out, homogeneous hyperenhancement
	1	Ia	4B	4C	earlier wash-in, later wash-out, heterogeneous hyperenhancement
inflammation	1	Ia	4B	4C	earlier wash-in, later wash-out, heterogeneous hyperenhancement
	1	IIa	4A	4B	
	1	IIa	4A	4B	earlier wash-in, later wash-out, heterogeneous hyperenhancement, enlarged enhancement extent
sclerosing adenosis	1	IIa	4A	4B	earlier wash-in, heterogeneous hyperenhancement, crab claw-like enhancement
	1	IV	4B	4C	earlier wash-in, heterogeneous hyperenhancement
fibrocystic mastopathy	1	Ib	4C	4B	heterogeneous isoenhancement
	1	IIb	4C	4B	
DCIS	3	IIa	4A	3	heterogeneous isoenhancement or hypoenhancement
	1	Ia	4B	4A	
IDC with mucinous carcinoma	1	IIa	4A	4A	earlier wash-in, later wash-out, homogeneous hyperenhancement

### 3.6 Impact of CEUS optimized BI-RADS Category on the Biopsy Decision

BI-RADS 4A was used as the threshold for biopsy: lesions classified as BI-RADS 3 were excluded from biopsy, while those classified as BI-RADS 4A, 4B, 4C, and 5 underwent biopsy. BI-RADS 4B was adopted as the biopsy threshold: lesions classified as BI-RADS 3 and 4A were not biopsied, whereas those classified as BI-RADS 4B, 4C, and 5 were biopsied. The corresponding biopsy rate, malignancy detection rate, and missed diagnosis rate of US and CEUS-optimized BI-RADS category under different biopsy thresholds are presented in **Table 8**.

**Table 8:** Impact of CEUS-optimized BI-RADS Category on biopsy for NMLs.

Biopsy threshold	Biopsy rate	Malignancy detection rate	Missed diagnosis rate
US-BI-RADS 4A or above	148 (100%)	77 (52.03%)	0 (0%)
US-BI-RADS 4B or above	68 (45.95%)	41 (60.29%)	36 (46.75%)
CEUS-optimized BI-RADS 4A or above	111 (75%)	74 (66.67%)	3 (3.90%)
CEUS-optimized BI-RADS 4B or above	85 (57.43%)	72 (84.71%)	5 (6.49%)

If the results of mammography and MRI were incorporated into the comprehensive imaging assessment, one patient with DCIS classified as CEUS-BI-RADS 3 underwent biopsy due to a mammography diagnosis of BI-RADS 4B, and another patient with DCIS classified as CEUS-BI-RADS 4A underwent biopsy following an MRI diagnosis of BI-RADS 4B.

## 4 Discussion

The conventional US features of NMLs are characterized by diverse morphologies, indistinct boundaries, and the absence of typical mass-occupying effects. These attributes reduce the diagnostic accuracy of the BI-RADS category for NMLs compared with mass-like lesions, rendering them a major challenge in conventional US diagnosis. According to the findings of Ko et al., all NMLs were classified as BI-RADS 4A or above based on their grayscale US features. If biopsies were performed on all of them, it would lead to considerable overuse of medical resources. This study aimed to construct a CEUS prediction model to optimize the BI-RADS category of NMLs and reduce unnecessary biopsies.

Previous studies (16, 17) have demonstrated that microcalcification is an independent risk factor for malignant NMLs. Drawing on the findings of Ko et al., NMLs with microcalcifications (type Ib and IIb) were classified as BI-RADS 4C in this study. These lesions exhibited a malignancy rate as high as 93.55%, significantly higher than lesions without microcalcifications. Therefore, we recommended that all NMLs with microcalcifications should be biopsied to confirm the pathological diagnosis. Conversely, the malignancy risk of NMLs without microcalcifications varied substantially. Given that conventional US alone was insufficient for accurate differentiation, the incorporation of CEUS was necessary to improve diagnostic accuracy. The CEUS prediction model established in this study provided excellent diagnostic performance for differentiating benign and malignant NMLs, with an area under the ROC curve (AUC) of 0.876 and an accuracy rate of 87.8%.

Univariate analysis of CEUS qualitative features in this study demonstrated that earlier wash-in, later wash-out, hyperenhancement, heterogeneous enhancement, enlarged enhancement extent, and crab claw-like enhancement were the main indicators of malignant NMLs. These were consistent with previous research findings (18, 19). Angiogenesis is a vital process for genesis and progression of the tumor, preceding the morphological alterations. Breast cancer cells secrete vascular endothelial growth factor (VEGF), which induces extensive neovascularization (20–22). The neovascular walls are structurally immature and highly permeable. Tumor blood vessels exhibit disorganized and tortuous architecture, with widened endothelial gaps. Consequently, the CEUS features of breast cancer were earlier wash-in, later wash-out, and hyperenhancement. There are heterogeneous regions such as necrosis, fibrosis, and hemorrhage within the tumor and disparate spatial distribution of blood vessels, resulting in heterogeneous enhancement on CEUS (23). Binary logistic regression analysis revealed that enlarged enhancement extent and crab claw-like enhancement were independent risk factors for malignant NMLs. Although both benign and malignant NMLs display indistinct boundaries and irregular shapes on US, malignant tumors grow in an invasive manner, with the peritumoral tissue hyperplasia area containing new blood vessels. The new blood vessels of malignant tumors often arrange in a radial pattern centered on the tumor (13, 24). Therefore, enlarged enhancement extent and crab claw-like enhancement can be observed on CEUS. In contrast, most benign NMLs exhibit non-invasive growth patterns, without abnormal neovascularization. The majority of benign NMLs don’t display the typical CEUS features associated with malignant lesions. However, a minority of benign NMLs demonstrate CEUS features similar to malignancy, due to imbalanced angiogenesis regulation or potential malignant biological behaviors. In this study, 13 benign NMLs were misdiagnosed as malignant after CEUS optimization, including atypical hyperplasia, intraductal papilloma, inflammation, sclerosing adenosis, and fibrocystic mastopathy. The underlying reason is that atypical hyperplasia and intraductal papilloma are precancerous lesions with active cellular proliferation and increased microvessel density. These features result in hyperenhancement on CEUS, which may lead to misdiagnosis (25–27). Inflammatory stimulation causes local vasodilation and increased blood flow within breast lesions. As the disease progresses, inflammatory cells infiltrate the surrounding tissue (28, 29), which may manifest as malignant CEUS features. Although sclerosing adenosis is a benign condition, it is often associated with capillary proliferation within the lobules and stroma, as well as stromal sclerosis and glandular distortion. These histopathological features can create a “pseudo-invasive” appearance, which may lead to misdiagnosis as malignancy (30). Fibrocystic mastopathy may present with malignant features on imaging, such as punctate hyperechoic foci, which are often attributable to micro-echo enhancement of the cyst wall and the posterior region. These findings can be easily misinterpreted as calcifications. In such cases, the local magnification function should be utilized, to carefully observe the internal structure of the mass and minimize the risk of misdiagnosis (31). In the CEUS prediction model, 5 malignant NMLs were misdiagnosed as benign, including DCIS and IDC with mucinous carcinoma. Ductal carcinoma *in situ* (DCIS) is characterized by the confinement of tumor cells within the mammary ducts, without invasion beyond the basement membrane. The ability of these tumor cells to stimulate the surrounding stromal vasculature is relatively limited. DCIS typically demonstrates low expression levels of VEGF, which contributes to a significantly reduced microvessel density (32). Moreover, CEUS is insensitive to microcalcifications, which represent a characteristic imaging feature of DCIS. This limitation may result in the misdiagnosis of certain DCIS lesions on contrast-enhanced imaging. Mucinous carcinoma is particularly prone to misdiagnosis on CEUS because it may exhibit benign features, such as expansive growth and relatively poor vascularity. This tendency is primarily attributable to the abundant extracellular mucin, pseudocapsule formation, and sparse blood vessels (33, 34).

To address the aforementioned issues of misdiagnosis, a multimodal imaging assessment can compensate for the limitations inherent to any single diagnostic modality. The diagnostic efficacy of mammography is significantly reduced in dense breasts, and the prevalence of breast density among Asian women is much higher than that in Western populations. A proportion of patients present primarily with localized breast symptoms, such as palpable masses or nipple discharge, and mammography is less accurate than ultrasound in differentiating between cystic and solid breast lesions. Mammography requires patients to be exposed to ionizing radiation, and breast compression is necessary during image acquisition, which is poorly tolerated by some patients due to discomfort. These factors have limited the application of mammography in this study. However, mammography demonstrates substantially higher sensitivity than CEUS for the detection of microcalcifications and is therefore more effective in identifying malignant lesions, such as DCIS. In addition, imaging findings should be interpreted in conjunction with clinical information and individual risk factors. For patients with high-risk characteristics, including advanced age or a family history of breast malignancy, lesions that appear benign on CEUS but suspicious on other imaging modalities should prompt a recommendation for biopsy. Conversely, the possibility of inflammatory lesions causing false-positive imaging findings should be carefully considered in patients presenting with localized pain, a history of trauma, or signs of inflammation.

If biopsy was performed on NMLs categorized as BI-RADS 4A or above based on conventional US, the biopsy rate reached 100% in this study. However, the malignancy detection rate was only 52.03%. It indicated that US-BI-RADS tended to overdiagnose a large number of benign NMLs, leading to unnecessary biopsies or surgical interventions. The BI-RADS categories of 148 NMLs were optimized by a CEUS prediction model in this study. Setting CEUS-optimized BI-RADS 4A as the biopsy threshold, the biopsy rate decreased to 75.00% while the malignancy detection rate increased to 66.67%. This approach avoided unnecessary biopsy for 37 lesions classified as CEUS-optimized BI-RADS 3, thereby reducing interventions for benign lesions. The missed diagnosis rate was only 3.90%. If CEUS-optimized BI-RADS category was used to guide breast biopsy, the detection rate of breast cancer increased and the malignant risk remained at a relatively low level. This provided valuable reference for making rational diagnosis and treatment decisions in clinical practice.

This study has some limitations. First, as a retrospective analysis, selection and recall biases were unavoidable. Second, sample capacity was small and diversity of pathology was relatively limited in this study. Therefore, multicenter studies with larger cohorts were warranted for validation. Third, only CEUS was used as the sole imaging modality, without integration of multimodal imaging approaches, such as elastography, mammography, or contrast-enhanced magnetic resonance imaging (MRI), which may have resulted in an incomplete assessment of lesion characteristics. In addition, CEUS itself has inherent limitations. Its interpretation relies heavily on the operator’s clinical experience and subjective judgment, and interobserver variability may compromise diagnostic consistency. Moreover, there is currently no universally accepted and standardized system for interpreting CEUS enhancement patterns; the evaluation criteria applied vary across studies, limiting the comparability of results. Finally, CEUS has limited sensitivity for detecting microcalcifications, which are often critical diagnostic clues for certain malignant lesions. This shortcoming may adversely affect early lesion detection and diagnostic accuracy.

## Conclusions

The CEUS prediction model offered an optimized approach to the BI-RADS category of NMLs. Implementing CEUS-optimized BI-RADS category to guide core needle biopsy may not only minimize unnecessary procedures but also improve the detection rate of malignant breast lesions.

## Data Availability

The raw data supporting the conclusions of this article will be made available by the authors, without undue reservation.
